# Depression and dental caries in US adults, NHANES 2015–2018

**DOI:** 10.1186/s12903-024-04288-5

**Published:** 2024-05-02

**Authors:** Zefeng Xie, Lingfang Shi, Libo He

**Affiliations:** 1 Department of Stomatology, Second Affiliated Hospital of Zhejiang University, Hangzhou, Zhejiang China; 2 Department of Stomatology, Xiaoshan Affiliated Hospital of Wenzhou Medical University, Hangzhou, Zhejiang China

**Keywords:** Depression, Untreated root caries, Untreated coronal caries, NHANES

## Abstract

**Purpose:**

This study aimed to investigate the link between depression and untreated dental caries among adults in the United States.

**Methods:**

Data were collected from the National Health and Nutrition Survey (2015–2018); respondents aged 20 years or older who completed a patient health questionnaire and underwent a comprehensive oral examination were included. Participants were categorized into three groups according to depressive symptoms as follows: those with no, mild, or moderate to severe depression. Data were weighted, and multiple potential covariates were included in the analysis to provide national estimates and account for the complex sample design. A multivariable weighted logistic regression model was performed to test the hypothesis that varying degrees of depression in American adults are associated with untreated dental caries. Subgroup analyses were performed based on age and gender after adjusting for potential covariates. A *P* value of <.05 was considered significant.

**Results:**

Among 8740 participants, the prevalence of untreated coronal and root caries was 20.50% and 12.92%, respectively. Moderate to severe depression was a significant risk factor (odds ratio, 1.25; 95% confidence interval, 1.09–1.66) for untreated root caries. The risk of untreated root caries increased by 87% in young adults (aged 20–44 years) and by 46% in women with moderate to severe depression. The suest analysis revealed that the impact of moderate to severe depressive disorder on untreated root caries was non-significantly different between the age subgroup (*p*=0.09) and sex subgroup (*p*=0.51). However, depression was non-significantly associated with untreated coronal caries (mild depression: OR, 1.07; 95% CI, 0.85–1.34; moderate to severe depression 1.06; 95% CI, 0.83–1.36; respectively).

**Conclusion:**

The results of this study suggested a significant association between moderate and severe depression and untreated root caries; however, the association with untreated coronal caries was non-significant. In the United States, moderate and severe depression in adults is associated with root caries.

## Introduction

Depression is a common disease in contemporary society that negatively impacts patients’ feelings, behaviors, and ways of thinking [1]. In addition, this condition is also a public health problem with a significant economic impact [2]. In 2019, the annual prevalence of a major depressive episode among US adults was 7.1% [3], and its relationship with other chronic diseases has been previously studied extensively [4–6].

Dental caries is also a common condition; according to statistics, more than 1 in 5 adults in the United States had untreated dental caries between 2017 and 2020 [7]. As material demands among individuals continue to rise across generations, the retention of teeth increases, leading to the anticipation of root caries emerging as a notable challenge in the field of dentistry [8]. Reports indicate that 14% of adults aged 20 and above in the United States experienced root caries between 2017 and 2020 [9]. According to research conducted in the United States, the exact prevalence of coronal and root caries was 17.9% and 10.1%, respectively [10].

In 2015, oral diseases and depression were among the top ten causes of disability worldwide [11], and an increasing number of researchers have begun to pay attention to the link between oral diseases and depression [12]. People with depression often experience symptoms of fatigue or lack of concentration [13], which can lead to an increased intake of carbohydrates and sugary foods, less brushing and flossing of the teeth, and fewer visits to the dentist. These symptoms ultimately increase the risk of dental caries [14]. Studies of associations between depression and oral health outcomes have increased over the years [15, 16]; however, few have explored the association between depressive symptoms and untreated dental caries [17]. Wiener noted in their report that the association between depressive symptoms and untreated coronal caries did not remain significant after tobacco use was added in 2017 [18]. Additionally, Aldosari also published a study in 2020 that linked depressive symptoms to mild periodontitis and a high number of missing teeth but did not explore the relationship between depressive states and untreated caries [19]. In contrast, Skokiewicz-Malinowska's findings indicated that among people 65 and older, the severity of depression increased with the number of missing teeth, number of cavities, and worsening dry mouth symptoms [20]. Lastly, Cademartori concluded in their study that the effect of dental caries on depression depends on oral health perceptions [21]. Therefore, in this study, we used a nationally representative sample to determine whether depression in American adults is associated with untreated dental caries. We hypothesized that varying degrees of depression are related to untreated root and coronal caries in adults. As no previous reports have examined the relationship between depression and untreated root and corona caries, our study fills a gap in the current literature.

## Methods

### Study population and design

This study used the 2015–2016 and 2017–2018 National Health and Nutrition Examination Survey (NHANES) data. Conducted by the US Center for Disease Control and Prevention, the NHANES survey is a cross-sectional study of non-institutionalized civilians selected to represent the US population of all ages. The survey includes detailed demographic and health questionnaires, laboratory assays, and clinical examination measures of health outcomes. Data is collected on an annual basis and has been publicly released in two-year intervals since 1999. NHANES uses a complex multistage sampling strategy to select a representative sample of the US population. Households are randomly selected within counties and eligible individuals within each household are selected to participate in the survey, with oversampling of certain subpopulations such as minorities to ensure adequate representation [22]. The NHANES methods and data collection procedures are described in detail at www.cdc.gov/nchs/nhanes.htm [23]. The total sample size included 19,225 participants in the two pooled cycles from the 2015 to 2018 National Health and Nutrition Examination Survey. We excluded participants with an age younger than 20 years (*n*=7937), incomplete overall oral examination (*n*=1032), lack of a measure of depression (*n*=805), edentulous jaw (*n*=672), absence of root caries (*n*=1), lack of education level records (*n*=6), smoking record (*n*=5), dentist visits (n=22) and diabetes (*n*=5). Ultimately, data from 8740 adults aged 20 years or older who completed the Patient Health Questionnaire and underwent a full oral examination with at least one sound permanent or primary tooth present (excluding the third molar) were extracted and included in the data analysis. The survey protocols for this study were approved by the National Center for Health Statistics Research Ethics Review Committee, and written informed consent was obtained from all participants or their representatives for data collection and analysis.

### Study variables

#### Outcome variable: untreated coronal and root caries

Licensed dentists performed NHANES oral health assessments following strict protocols, and data were recorded using a separate dental recorder. After rinsing the mouth, food debris was removed with 2×2-in sponges. All teeth (except the third molars) were dried with air when needed and examined using a no. A #23 dental explorer and surface-reflecting mirrors. The teeth of each participant were examined in standardized order, and the presence of untreated coronal and root caries was recorded. The diagnostic criteria for untreated coronal caries included the presence of gross cavitation; a deep pit or fissure with softness at the base or an adjacent opacity indicating undermining or demineralization; white spots or subsurface demineralization found to be soft on probing with an explorer; proximal caries diagnosed using similar criteria as for deep pits, fissures, or smooth surfaces; breaks in enamel or subsurface shadowing on proximal tooth surfaces; and loss of translucency identified via transillumination on proximal surfaces of anterior teeth only. Untreated root caries were considered present if active lesions were found on exposed root surfaces after drying including yellow or orange, tan, or light brown lesions, lesions soft on probing, or lesions with cavitation below the cementoenamel junction. The primary outcome variable was the presence of untreated coronal caries or root caries (yes or no). The prevalence of untreated coronal or root caries was defined as the percentage of participants with at least one untreated coronal or root caries. The process for acquiring the outcome variables can be found at www.cdc.gov/nchs/nhanes.

#### Exposure variable: depressive symptoms

The primary exposure variable was the category of depressive symptoms (none, mild, or moderate to severe). Severity was determined based on answers provided during the interview utilizing the Patient Health Questionnaire-9 (PHQ-9). The PHQ-9 is a screening tool consisting of nine questions specifically designed to assess depression. Participants were questioned about their depressive status during the previous two weeks and asked to express how troubled they were by the situation presented. The answers were scored as “not at all” (0), “several days” (1), “more than half of the time” (2), and “nearly every day” (3). The scores for all nine questions were summed. The total score ranged from 0 to 27. A total score of 0–4 represents no depressive symptoms (reference group); a total score of 5–9 indicates mild depressive symptoms; a total score of 10 and higher indicates moderate to severe depressive symptoms. A PHQ-9 score of 10 has been recommended in the literature as the cut-off point for determining major depression [24].

#### Other independent variables

The following independent variables were considered as potential covariates in this analysis.

Age was a continuous variable in this study, and participant sex was categorized as male or female, while race was classified as Caucasian, Mexican American, Non-Hispanic Black, or other race. Education level was grouped as ≤ high school or more than high school. Family income level was determined using the family income-to-poverty ratio (PIR) and categorized as < 1.3, 1.3–3.5, and >3.5 [25]. Smoking status was self-reported and classified as never smoked, current smoker, or former smoker. Participants' diabetes mellitus status was self-reported and categorized as either yes or no. Filled root caries and filled coronal caries were categorized as yes or no based on history of previous restorations. The number of teeth was recorded (1–28, excluding the third molars). Recent dental visits were categorized as within the past year or more than one year. CRP levels were categorized into three levels (<1.0 mg/L; 1.0–3.0 mg/L; and > 3.0 mg/L) according to CDC/AHA recommendations [19]. Total sugar intake in the first 24-h dietary recall data was categorized by tertiles as low, middle, and high. All potential covariates were obtained directly from the dataset of NHANES.

### Statistical analysis

All statistical analyses were performed using the Stata 15 Software (Stata Corp LLC, College Station, TX, USA). Associations were presented using odds ratios [OR] with 95% confidence intervals [CI], and p < 0.05 was considered significant. Survey methods were employed to analyze the intricate sampling structure of the NHANES dataset. National-level results were generated using four-year survey sample weights. Although the 2015-2016 and 2017-2018 NHANES datasets are independent, according to the formula provided by the NHANES dataset, new multi-year sample weights can be calculated by dividing the two-year sample weights by the number of two-year periods considered in the analysis. Categorical variables were assessed using chi-square tests, while continuous variables were evaluated using analysis of variance tests to examine differences between participants categorized by their depression status.

After adjusting for covariates, we constructed six logistic regression models using weighted data to explore the association between depression status and untreated dental caries. These models were divided into two groups: three for root caries (Models I, II, III) and three for coronal caries (Models IV, V, VI).

Model I was unadjusted. Model II was adjusted for age, sex, race, education, family PIR, diabetes, untreated coronal caries, filled root caries, tooth count, CRP, and sugar intake. Model III extended Model II by including smoking and dentist visits.

Model IV remained unadjusted. Model V mirrored Model II's adjustments but substituted untreated coronal caries and filled root caries with untreated root caries and filled coronal caries, respectively. Model VI extended Model V by including smoking and dentist visits.

Additionally, the same covariates were adjusted for subgroup analysis stratified by age and sex in the model III and model VI. Responses of "Refused," "Don't know," and "Missing" among the adjusted variable were combined into a single missing category. This missing category of the variable was then incorporated into the multifactorial logistic regression to ensure that its potential impact on the outcomes was considered. All ORs for the two models were reported. The seemingly unrelated estimation (suest) method was employed to assess the differences in the impact of depression across subgroups, allowing for the comparison of coefficients between subgroups by performing the adjusted Wald test to evaluate the statistical differences in the effect of depression.

## Results

### Baseline characteristics

Data for the 8740 participants were all taken from the NHANES database. Approximately 203 million adults were identified by weighing the samples. The average age of the study sample was 47.34 ± 0.41 years, and 48.64% of participants were men. Among the participants, 76.22% were depression-free, 16.08% had mild depression, and 7.70% had moderate to severe depression. The prevalence of untreated root and coronal caries in adults was 12.92% and 20.50%, respectively, and individuals with moderate to severe depression were more likely to be young, female, non-Hispanic Black, diabetic, and current smokers. They also have lower levels of education and income. They intake more sugar, have fewer dental caries restorations and higher CRP levels, have periodontal diseases, have more untreated dental caries, and have fewer teeth. In addition, these individuals had fewer dental visits (Table 1).

**Table 1:** Characteristics of participants (≥ 20 years of age) stratified by depression status category (NHANES 2015–2018)

**Characteristic**	**Full sample N(weighted%) or mean± SEs**	**Depression status category** **N (weighted %) or mean ± SEs**			***P* value**
		**No**	**Mild**	**Moderate/severe**	
**participants number**	8740	6572(76.22)	1463(16.08)	705(7.7)	
**Sociademographic**					
**1.Age (years)**	47.34±0.41	47.57±0.42	47.00±0.67	45.86±0.98	0.07
**2.Sex**					<0.001
Female	4480(51.36)	3190 (48.9)	855(57.41)	435(63.07)	
Male	4260 (48.64)	3382 (51.1)	608(42.59)	270(36.93)	
**3.Race/ethnicity**					0.71
Caucasian	2981(64.34)	2170(64.5)	535(63.96)	276(63.61)	
Mexican American	2379(15.43)	1771(15.26)	414(16.26)	194(15.34)	
Non-Hispanic Black	1926(10.92)	1450(10.72)	321(11.46)	155(11.76)	
Other races	1454(9.31 )	1181(9.52)	193(8.32)	80(9.29)	
**4.Education**					<0.001
> High school	5069 (65.22)	3967(67.5)	752(58.31)	350(57.09)	
≤High school	3671(34.78)	2605(32.5)	711(41.69)	355(42.91)	
**5.Family PIR**					<0.001
>3.5	2398 (44.76)	2019(49.28)	278(32.21)	101(26.04)	
1.3-3.5	3167 (36.2)	2378(35.18)	557(41)	232(36.28)	
<1.3	2230(19.04)	1458(15.54)	480(26.79)	292(37.68)	
**6.Smoking status**					<0.001
Never smoking	5176(58.02)	4113(61.98)	764(48.25)	299(39.22)	
Current smoking	1563(16.86)	980(13.32)	337(23.9)	246(37.19)	
Former smoking	2001(25.12)	1479(24.7)	362(27.85)	160(23.59)	
**7.Diabetes mellitus**					<0.001
No	7498(89.34)	5716(90.22)	1209(87.07)	573(85.35)	
Yes	1,242(10.66)	856(9.78)	254(12.93)	132(14.65)	
**8.Filled coronal caries**					<0.001
No	1700(16.17)	1244(15.28)	298(18.36)	158(20.41)	
Yes	7040(83.83)	5328(84.72)	1165(81.64)	547(79.59)	
**9.Filled root caries**					0.57
No	8330 (95.20)	6272(95.25)	1392(94.7)	666(95.67)	
Yes	410 (4.80)	300(4.75)	71(5.3)	39(4.33)	
**10.Number of teeth**	24.61±0.13	24.94±0.13	23.89±0.21	23.17±0.44	<0.001
**11.Dental visit**					<0.001
Within past year	4821(60.56)	3722(62.54)	764(55.66)	335(51.19)	
More than one year	3919(39.44)	2850(37.46)	699(44.34)	370(48.81)	
**12.CRP(mg/L)**					<0.001
< 1	2450(31.26)	1917(32.61)	381(28.75)	152(23.16)	
1-3	2827(34.66)	2201(35.57)	423(31.22)	203(32.85)	
>3	2995(34.08)	2092(31.82)	584(40.03)	319(43.99)	
**13.Total sugar intake**					0.007
Low	2792(32.54)	2095(31.89)	463(34.29)	234(35.35)	
Middle	2792(32.31)	2156(33.09)	446(31.36)	190(26.44)	
High	2797(35.15)	2055(35.02)	496(34.35)	246(38.21)	
**14.Having untreated coronal caries**					<0.001
No	6567 (79.5)	5059(81.51)	1053(74.86)	455(69.25)	
Yes	2173 (20.5)	1513(18.49)	410(25.14)	250(30.75)	
**15.Having untreated root caries**					<0.001
No	7366(87.08)	5657(88.88)	1186(83.72)	523(76.31)	
Yes	1374(12.92)	915(11.12)	277(16.28)	182(23.69)	

*Abbreviations* Family PIR, family income-to-poverty ratio. Continuous variables are expressed as mean ± SEs (standard errors). Categorical variables are expressed as percentages

Family PIR categories had 945 missing observations. CRP categories had 468 missing observations. Total sugar intake categories had 359 missing observations

### Relationship between depression and untreated root caries

The unadjusted results indicate that depressed participants had a higher risk of root caries than those without depression (Table 2). In model II, the moderate to severely depressed participants were 50% more likely to develop root caries than those without depression (OR, 1.50; 95% CI, 1.20–1.87). Even after adjusting for covariates that increased dental visits and smoking status in model III, the moderate to severely depressed participants were 25% more likely to develop root caries than those without depression (OR, 1.25; 95% CI, 1.09–1.66) (Table 2). The comparison of results between models II and III also suggested that the two covariates of dental visits and smoking status had minimal effect on our study.

**Table 2:** Weighted multivariable logistic regression analysis of depression with untreated root caries among adults (≥ 20 years of age) in the USA (NHANES 2015–2018)

**Characteristics**	**Model I**	**Model II**	**Model III**
	OR (95% CI)	OR (95% CI)	OR (95% CI)
**Depression**			
** No**	**Ref**	**Ref**	**Ref**
** Mild**	1.55(1.31-1.85)***	1.10(0.89-1.36)	1.04(0.84-1.29)
** Moderate/Severe**	2.48(1.96-3.15)***	1.50(1.20-1.87)**	1.25(1.09-1.66)**
** Age (years)**		1.01(1.00-1.01)*	1.01(1.00-1.02)**
**Sex**			
** Female**		**Ref**	**Ref**
**Male**		1.31 (1.10–1.57)**	1.21 (1.01–1.45)*
**Race/ethnicity**			
** Caucasian**		Ref	Ref
** Mexican American**		0.61 (0.45-0.83)**	0.64 (0.48-0.88)**
** Non-Hispanic Black**		0.98 (0.73-1.33)	1.01 (0.74-1.39)
** Other races**		0.97 (0.63-1.50)	1.01 (0.64-1.57)
**Education**			
** > High school**		**Ref**	**Ref**
** ≤ High school**		1.16 (0.95-1.42)	1.09 (0.89-1.35)
**Family PIR**			
** > 3.5**		Ref	Ref
** ≥ 1.3, ≤ 3.5**		1. 79 (1.35-2.37)***	1. 62 (1.21-2.18)**
** < 1.3**		2.05 (1.59-2.65)***	1.77 (1.33-2.34)***
**Diabetes**			
** No**		Ref	Ref
** Yes**		0.99 (0.74-1.33)	1.05 (0.77-1.43)
**Having untreated coronal caries**			
** No**		Ref	Ref
** Yes**		9.96 (7.45-13.32)***	8.75 (6.65-11.52)***
**Filled root caries**			
** No**		Ref	Ref
** Yes**		2.75 (1.67-4.54)***	2.79 (1.65-4.73)***
** Number of teeth**		0.89 (0.87-0.91)***	0.90 (0.88-0.91)***
**CRP(mg/L)**			
** < 1**		0.71 (0.55-0.92)*	0.73 (0.56-0.94)*
** 1-3**		Ref	Ref
** >3**		1.25 (1.01-1.55)*	1.23 (0.98-1.54)
**Total sugar intake**			
** Low**		0.82 (0.61-1.11)	0.83 (0.61-1.13)
** Middle**		0.86 (0.71-1.05)	0.89 (0.72-1.09)
** High**		Ref	Ref
**Smoking status**			
** Never smoking**			Ref
** Current smoking**			1.72 (1.35-2.18)***
** Former smoking**			1.06 (0.89-1.27)
**Dental visit**			
** Within past year**			Ref
** More than one year**			1.56 (1.24-1.97)***

Model I was adjusted for: None. Model II was adjusted for: age,sex,race,edu,familypir,diabetes, untreated coronal caries, filled root caries, number of teeth, CRP, and total sugar intake. Model III was adjusted for: age,sex,race,edu,familypir,diabetes, untreated coronal caries, filled root caries, number of teeth, total sugar intake, CRP,smoking, and dental visit

*Abbreviations CI* confidence interval, *OR* odds ratio, *Ref* reference, *Family PIR,* family income-to-poverty ratio

^*^p< .05

^**^p< .01

^***^p< .001

### Subgroup analysis for the association of depression with untreated root caries

After adjusting for all covariates, younger adults (20–44 years) with moderate to severe depression had an 87% higher risk of untreated root caries (OR, 1.87; 95% CI, 1.29–2.71) compared to those without depression. This association was not observed among middle-aged and older adults (aged ≥45). Women with moderate to severe depression had a 46% higher risk of untreated root caries (OR, 1.46; 95% CI, 1.05–2.04) compared to those without depression. This association was not observed in men.

However, the suest analysis indicated that the impact of moderate to severe depression on untreated root caries did not exhibit significant differences across age groups (*p*=0.09) or sex subgroups (*p*=0.51) (Table 3).

**Table 3:** Age subgroup analysis and sex subgroup analysis for the association between depression and untreated root caries among adults (≥ 20 years of age) in the USA (NHANES 2015–2018)

**Subgroup**	**Age**			**Sex**		
	20≤Age≤44	45≤Age		women	men	
**N(weighted %)**	3725 (45.60)	5015 (54.40)		4480(51.36)	4260(48.64)	
**Characteristics**	OR (95% CI )	OR (95% CI )		OR (95% CI )	OR (95% CI )	
**Depression**			**P for interaction**			**P for interaction**
**No**	**Ref**	**Ref**		**Ref**	**Ref**	
**Mild**	0.95 (0.67-1.35)	1.11 (0.81-1.53)		0.94 (0.64-1.39)	1.14 (0.83-1.56)	
**Moderate/Severe**	1.87 (1.29-2.71)**	1.10 (0.76-1.59)	0.09	1.46 (1.05-2.04)*	1.19 (0.78-1.83)	0.51

Adjusted for: age,sex,race,edu,familypir,smoking,diabetes, untreated coronal caries, filled root caries, number of teeth, dental visit, CRP, and total sugar intake.

*Abbreviations CI* confidence interval, *OR* odds ratio, *Ref* reference, *Family PIR* family income-to-poverty ratio

^*^p< .05

^**^p< .01

^***^p< .001

### Association of depression with untreated coronal caries

The unadjusted results indicated that depressed participants were more likely to have untreated coronal caries than those without depression (mild depression: OR, 1.48; 95% CI, 1.25–1.76; moderate to severe depression 1.96; 95% CI, 1.54–2.49; respectively) (Table 4). After adjustment for covariates in models V and VI, the significant association between mild and moderate to severe depression and untreated coronal caries disappeared. Depressed participants were not found to have an increased likelihood of developing coronal caries compared to non-depressed participants (Table 4). Additionally, the comparison between models V and VI indicated that dental visits and smoking status, as covariates, had minimal impact on our study.

**Table 4:** Weighted multivariable logistic regression analysis of depression with untreated coronal caries among adults (≥ 20 years of age) in the USA (NHANES 2015–2018)

**Characteristics**	**Model IV**	**Model V**	**Model VI**
	**OR (95% CI)**	OR (95% CI)	**OR (95% CI)**
**No**	**Ref**	**Ref**	**Ref**
**Mild**	1.48 (1.25-1.76)***	1.13 (0.90-1.41)	1.07 (0.85-1.34)
**Moderate/Severe**	1.96 (1.54-2.49)***	1.18 (0.92–1.51)	1.06 (0.83–1.36)
**Age (years)**		0.98 (0.97–0.98)***	0.98 (0.98–0.99)***
**Sex**			
** Female**		**Ref**	**Ref**
** Male**		1.26 (1.03–1.54)*	1.13 (0.93–1.36)
**Race/ethnicity**			
** Caucasian**		Ref	Ref
** Mexican American**		1.13 (0.86-1.49)	1.17 (0.90-1.52)
** Non-Hispanic Black**		1.64 (1.25-2.16)**	1.68 (1.28-2.20)**
** Other races**		1.00 (0.73-1.39)	1.05 (0.76-1.43)
**Education**			
** > High school**		**Ref**	**Ref**
** ≤ High school**		1.74 (1.45-2.09)***	1.59 (1.35-1.89)***
**Family PIR**			
** ≥ 3.5**		Ref	Ref
** ≥ 1.3, <3.5**		1. 81 (1.44-2.28)***	1. 58 (1.24-2.01)**
** < 1.3**		2.36 (1.91-2.91)***	1.93 (1.54-2.42)***
**Diabetes**			
** No**		Ref	Ref
** Yes**		1.08 (0.85-1.35)	1.11 (0.88-1.40)
**Having untreated root caries**			
** No**		Ref	Ref
** Yes**		9.18 (6.87-12.27)***	8.16 (6.19-10.74)***
**Filled coronal caries**			
** No**		Ref	Ref
** Yes**		0.85 (0.73-0.99)*	0.94 (0.80-1.10)
** Number of teeth**		0.97 (0.95-0.99)**	0.97 (0.95-0.99)*
**CRP(mg/L)**			
** < 1**		1.10 (0.88-1.38)	1.15 (0.92-1.45)
** 1-3**		Ref	Ref
** >3**		1.22 (1.01-1.47)*	1.19 (1.00-1.42)*
**Total sugar intake**			
** Low**		0.75 (0.62-0.92)**	0.76 (0.62-0.93)**
** Middle**		0.82 (0.66-1.02)	0.84 (0.67-1.05)
** High**		Ref	Ref
**Smoking status**			
** Never smoking**			Ref
** Current smoking**			1.63 (1.34-2.01)***
** Former smoking**			1.09 (0.86-1.37)
**Dental visit**			
** Within past year**			Ref
** More than one year**			2.17 (1.79-2.63)***

Model IV was adjusted for: None. Model V was adjusted for: age,sex,race,edu,familypir,diabetes, untreated coronal caries, filled root caries, number of teeth, CRP, and total sugar intake. Model VI was adjusted for: age,sex,race,edu,family PIR,diabetes, untreated root caries, filled coronal caries, number of teeth, CRP, total sugar intake, smoking, and dental visit

*Abbreviations CI* confidence interval, *OR* odds ratio, *Ref* reference, *Family PIR*, family income-to-poverty ratio

^*^p< .05

^**^p< .01

^***^p< .001

### Subgroup analysis for the association of depression with untreated coronal caries

Separate subgroup analyses showed no significant association between depression and the prevalence of untreated coronal caries, regardless of age or sex (Table 5).

**Table 5:** Age subgroup analysis and sex subgroup analysis for the association between depression and untreated coronal caries among adults (≥ 20 years of age) in the USA (NHANES 2015–2018)

**Subgroup**	**Age**			**Sex**		
	20≤Age≤44	45≤Age		women	men	
**N(weighted %)**	3725 (45.60)	5015 (54.40)		4480(51.36)	4260(48.64)	
**Characteristics**	OR (95% CI )	OR (95% CI )		OR (95% CI )	OR (95% CI )	
**Depression**			**P for interaction**			**P for interaction**
**No**	**Ref**	**Ref**		**Ref**	**Ref**	
**Mild**	1.01 (0.75-1.36)	1.17 (0.82-1.66)		1.16 (0.80-1.69)	0.96 (0.73-1.27)	
**Moderate/Severe**	0.95 (0.65-1.39)	1.15 (0.80-1.67)	0.47	1.23 (0.91-1.66)	0.87 (0.61-1.25)	0.11

Adjusted for: age,sex,race,edu,family PIR,smoking,diabetes, untreated coronal caries, filled root caries, number of teeth, dental visit, CRP, and total sugar intake.

*Abbreviations CI* confidence interval, *OR* odds ratio, *Ref* reference, *Family PIR*, family income-to-poverty ratio

^*^p< .05

^**^p< .01

^***^p< .001

## Discussion

Our study investigated the relationship between untreated dental caries and depression using nationally representative NHANES data. The findings indicate a link between depression and untreated root caries, highlighting a significantly higher risk of root caries among adults with moderate to severe depression. depression is non-significantly associated with untreated coronal caries.

Several studies have investigated the relationship between depression and untreated dental caries. Wiener et al found no association between categories of depressive symptoms and untreated coronal dental caries after adjusting for tobacco use and other socio-economic variables in the logistic regression model [18]. Aldosari et al discovered that the link between the severity of depressive symptoms and dental caries disappeared after accounting for confounding factors [19]. However, Guo identified a relationship between decayed, missing, filled teeth index (DMFT) and depressive symptoms in Chinese adolescents [26]. Two systematic reviews and meta-analyses suggest an association between depression and dental caries [27, 28]. Except for Wiener, no other studies have differentiated between crown caries and root caries, and there is a lack of similar studies in the literature on the association between root caries and depression. Discrepancies between our results and those in the literature may stem from variations in depression assessment methods, differences in the studied populations, or disparities in oral examination protocols and diagnoses.

Dental caries are multifactorial diseases related to the host, environment, bacteria, and time [29, 30]. Bacteria use carbohydrates in the oral environment to produce acidic products that demineralize the tooth root surface, resulting in root caries. First, depression negatively affects the patients’ feelings, thinking, and behavior. Patients with depression, especially those with moderate to severe depression, often have symptoms of malnutrition and overeating and consume large amounts of high-sugar foods[31–33]. Second, the oral health control of patients with depression is inadequate[34]; As the symptoms of depression worsen, people with depression need to invest more energy and economy in the treatment and care of depression, the importance of oral health gradually decreases, and the investment in oral care will also decrease. This could also explain why adults with mild depression do not have a significantly increased risk of root caries, but adults with moderate to severe depression do [35, 36]. The third possible cause is dry mouth. Depression-related physiological responses may reduce salivary production owing to sympathetic stimulation[37, 38]. However, antidepressants, which are often prescribed for long-term use, can also decrease oral saliva production, especially in those with anticholinergic effects [39, 40]. Fourth, studies have shown that depression often leads to a decrease in immunity, facilitating the reproduction of oral flora, and the central role of bacteria in the development of caries has been shown [41, 42].

However, our study found that depression does not increase the risk of untreated coronal caries. While root and coronal caries share similar risk factors, our investigation yielded contrasting results, which we attribute to subtle differences between the two types. Despite individuals with identical dentition theoretically facing equal probabilities of developing both coronal and root caries due to external and internal bodily conditions, oral hygiene plays a crucial role in caries control. The coronal area is easier to clean compared to the root area, which requires more attention. Consequently, with the same level of effort, the risk of root caries rises. As depression worsens, oral hygiene attention declines, leading to a disparity between root and coronal caries. Moreover, the rougher root surface is more prone to microbial attachment, resulting in more caries compared to the enamel on the crown surface [43, 44]. The root surface, consisting of cementum and dentin, is more susceptible to dental caries than the coronal enamel [45].

Furthermore, periodontal disease significantly links root caries and depression. Depression can exacerbate periodontal disease development as individuals with depression often exhibit negative attitudes towards life and engage in health-risk behaviors like smoking, alcohol consumption, and poor oral hygiene, known contributors to periodontal disease [16, 46, 47]. Alterations in the hypothalamic-pituitary-adrenal axis activity in depressed individuals lead to adrenal and cortisol interference, immune dysregulation, and increased secretion of pro-inflammatory cytokines such as IL-6, TNF-α, and C-reactive protein [48–50]. Psychological stress and cortisol level changes are suggested to heighten the risk of periodontal disease [51].

Studies have demonstrated a positive association between periodontal disease and root caries. As periodontal disease advances, gingival atrophy and increased root surface exposure in the oral cavity elevate the risk of root caries [43, 44]. Receding gums and root surface exposure to oral aerobic flora are common in periodontitis, in which the buffering effect of saliva neutralizes pH and alters the root environment, fostering the growth of cariogenic bacteria. This process may elucidate the role of periodontitis in root caries formation.

In addition, our results indicate that neither age nor sex significantly affected the correlation between depression and dental caries. Additionally, as covariates dental visits or smoking had minimal impact on our study. Studies by Dahl et al. have shown that depression can affect oral health in older adults independently of other factors, such as smoking and reduced tooth count [52], which is consistent with our findings. Other previous studies have mostly looked at the relationship between age, sex, smoking, dental visits and depression, or dental caries, but few have examined the impact of these factors on the relationship between dental caries and depression [14, 53, 54]. Therefore, further research is needed to explore the nature of this relationship.

This study had a few limitations. First, its cross-sectional design limited the ability to determine a causal relationship between depression and untreated root and coronal caries. Second, the NHANES database only shows the prevalence of root caries and excludes the number and severity of root caries, which limits further study on the association of depression with root caries. Third, depression was determined using the PHQ-9 scores; while this questionnaire is an established and validated tool for assessing depression, there are still some deviations from a true clinician’s diagnosis [55].

However, our study, which relied on the NHANES database, significantly increased the validity of the findings and extended the findings to adults across the United States. At the same time, this study is the beginning of a series aimed at exploring the direct relationship between depression and root caries. The ultimate purpose of this study was to provide a reference and theoretical basis for the clinical screening of depression. Therefore, additional studies are required to explore the association between depression and root caries. Given the findings of this study, we believe it is justified to promote integrated oral and mental healthcare services.

## Conclusion

The findings of this study suggest that depression was non-significantly associated with untreated coronal caries. However, a significant association was found between moderate and severe depression and untreated root caries. These results indicate that in the United States, moderate and severe depression in adults is associated with root caries.

## Data Availability

NHANES data are available for public download through the website (http://wwwn.cdc.gov/nchs/nhanes/). The code files for processing this study are available from the corresponding author upon request.

## Abbreviations

NHANES: National Health and Nutrition Survey
CEJ: Cementoenamel junction
PHQ-9: Patient Health Questionnaire-9
PIR: Poverty income ratio
